# Early Access to Sign Language Boosts the Development of Serial Working Memory in Deaf and Hard-of-Hearing Children

**DOI:** 10.3390/bs15070919

**Published:** 2025-07-07

**Authors:** Brennan P. Terhune-Cotter, Matthew W. G. Dye

**Affiliations:** 1 Laboratory for Language and Cognitive Neuroscience, San Diego State University, San Diego, CA 92182, USA; bterhunecotter@sdsu.edu; 2 NTID SPACE Center, Rochester Institute of Technology, Rochester, NY 14623, USA

**Keywords:** deaf and hard of hearing, American Sign Language, working memory, cognitive development, longitudinal, preteen children

## Abstract

Deaf and hard-of-hearing (DHH) children are often reported to show deficits on working memory (WM) tasks. These deficits are often characterized as contributing to their struggles to acquire spoken language. Here we report a longitudinal study of a large (N = 103) sample of DHH children who acquired American Sign Language (ASL) as their first language. Using an n-back working memory task, we show significant growth in WM performance across the 7–13-year-old age range. Furthermore, we show that children with early access to ASL from their DHH parents demonstrate faster WM growth and that this group difference is mediated by ASL receptive skills. The data suggest the important role of early access to perceivable natural language in promoting typical WM growth during the middle school years. We conclude that the acquisition of a natural visual–gestural language is sufficient to support the development of WM in DHH children. Further research is required to determine how the timing and quality of ASL exposure may play a role, or whether the effects are driven by acquisition-related corollaries, such as parent–child interactions and maternal stress.

## 1. Introduction

Working memory (WM) refers to the human ability to maintain information in an active state while performing cognitive tasks with that information. The most widespread theory of WM, multicomponent *WM*, holds that WM consists of multiple interrelated processing systems controlled by a central executive, which decides the importance and therefore the availability of the representations stored within these processing systems (Baddeley & Hitch, 1974; see Cowan, 2017 for a review of WM). The processing systems, which vary across theoretical frameworks but generally include a phonological buffer, a visuospatial buffer, and an episodic buffer, are distinguished by the computational processes by which they store and perform operations upon representations. Therefore, a task’s demands determine which processing systems are the most effective.

The phonological buffer is also called phonological short-term memory (STM). Whereas WM refers to the entire process of the encoding, storage, and retrieval of relevant information in many different forms, phonological STM refers specifically to the storage of serially ordered information as a phonological code in the phonological buffer (Hall & Bavelier, 2010). The phonological and serial nature of STM means it is uniquely optimized for the representation and manipulation of spoken linguistic units. The phonological and grammatical content of spoken language is, by necessity of the means of production and comprehension in the auditory–verbal modality, arranged sequentially in temporal chunks of information. Temporal order information is key for parsing spoken language, as well as other nonverbal cognitive skills, such as cause/effect understanding (Hall & Bavelier, 2010). STM interacts with the articulatory loop, which is a WM process that enables speakers to maintain verbal information in their memory by covertly or overtly reciting the information (Hall & Bavelier, 2010).

### 1.1. Working Memory in DHH Individuals

Many studies have investigated how executive functions operate in deaf and hard-of-hearing (DHH) children and young adults; they have often found that DHH individuals are at risk of diminished executive function relative to their hearing peers (e.g., Jamsek et al., 2022; Kronenberger et al., 2014; Marschark et al., 2016; McCreery & Walker, 2022). However, studies of signing DHH children have typically found that early access to sign language input appears to be a protective factor (e.g., Hall et al., 2018; Goodwin et al., 2022; Delcenserie et al., 2024). Studies which specifically investigate STM often aim to determine whether STM differences explain, or are explained by, the difficulties that DHH individuals may experience in acquiring spoken language. These studies commonly use serial recall tasks, in which participants are asked to recall spoken or visually presented lists of items (such as digits) in the order in which they were presented.

Cleary et al. (2001) compared 45 DHH cochlear implant (CI) users with 45 typically hearing controls, all aged 8–9 years, as they completed a serial recall task with patterned shapes, auditory labels, or both. They found that the DHH group achieved shorter span scores overall and did not benefit from the addition of auditory labels as much as the typically hearing group did. The shorter forward digit spans in such DHH children has also been positively correlated with spoken and written language outcomes (Pisoni & Cleary, 2003; Pisoni & Geers, 2000). While the analyses in those papers did not determine causal effects, the authors concluded that such differences in verbal WM capacities could explain the interindividual variability in speech and language outcomes among this population of CI users. Pisoni and Cleary (2003) also compared children in oral-only and total-communication environments and found that the oral-only children had longer forward digit spans, which they interpreted as evidence that spoken language exposure supports the development of phonological STM.

DHH people who primarily use a natural signed language to communicate often display a disadvantage on serial recall tasks requiring recall of *ordered* sequences, but not free recall tasks, as compared to their hearing peers (Bavelier et al., 2008). DHH signers exhibit a sign-based encoding and rehearsal of items into their STM, as evidenced by the phonological errors they make in serial recall tasks, which cohere with the phonological parameters of their signed language (Bellugi et al., 1974). Signed languages exhibit coarser temporal information and longer articulation times as compared to spoken languages, which have been cited as the reasons for serial recall differences between signers and speakers.

Marshall et al. (2015) studied WM abilities in DHH children with DHH parents (henceforth, DoD children), DHH children with hearing parents (henceforth, DoH children), and typically hearing children aged 6–11 years. They administered two WM tasks: the Spatial Span Task, which requires the participant to tap a sequence of blocks in the same or reverse order as shown by the examiner, and the Odd-One-Out task, which requires the participant to recall the location of one shape which differed from two other shapes shown in a grid. They found that on both tasks, the typically hearing and DoD children performed comparably, whereas the typically hearing children outperformed the DoH children. They also found that when controlling for age and nonverbal reasoning, the children’s expressive vocabulary (measured with the Expressive One Word Picture Vocabulary Test) predicted their WM task performance. They concluded that early and robust language experience, rather than deafness, impacts the development of nonverbal executive-loaded WM.

### 1.2. The N-Back Task: A Measure of WM

The n-back task is a test of the STM for serial sequences, which demands more executive processing than typical serial span tasks. In an n-back task, the participant views a continuous stream of items belonging to a delimited group (such as digits, letters, or images) and responds whenever the current item is identical to what appeared *n* items before. For a 1-back task, it is the immediately prior item (meaning two identical items appeared consecutively); for a 2-back task, the items are separated by one item; and so on, with greater values of *n* requiring greater executive and storage capacity.

Unlike serial span tasks, the n-back task is a continuous performance measure during which the participant cannot know which stimuli are relevant until after they disappear. Therefore, decisions about the relevance of the stimuli must be made while they are held in the WM. The dynamic format of the n-back task could be expected to attenuate reliance on verbal chunking and the rote rehearsal of information, which are the heuristic strategies theorized by Hall and Bavelier (2010) to give speakers an advantage over signers on serial span tasks requiring the ordered recall of fixed spans. Therefore, the n-back task can be considered a measure of the serial STM capacity, which minimizes the use of heuristic shortcuts commonplace to traditional serial span tasks.

The n-back task requires the participant to both maintain the serial order information in their STM and dynamically recall the items in serial order according to the task-based goals. For the 2-back test, participants must suppress irrelevant information (the immediately prior item) while maintaining it in their memory as the subsequent 2-back comparison item. Thus, the *n*-back task, particularly at higher ns, incorporates what Cowan (2017) calls attention-control WM, which refers to using attention to preserve the task-relevant information and inhibit the irrelevant information. These attention-control mechanisms are domain-general, and distinct from passive buffers, such as STM, which are domain- and modality-specific (Engle et al., 1999).

There have not been any behavioral studies that have studied WM in DHH people using visual *n*-back tasks. However, the n-back task is frequently used as a WM task in neuroimaging studies due to its simplicity and easy parameterization of difficulty (by increasing the *n*) (Pelegrina et al., 2015). Cardin et al. (2018) scanned DHH adults with a functional MRI as they performed 2-back tasks with point-light display stimuli, which were either linguistic (British Sign Language [BSL] signs) or nonlinguistic (moving objects). They aimed to determine whether the observed differences in neural recruitment during the WM tasks were due to the linguistic nature of the stimuli, the variation in the language experience of DHH people, or truly due to the absence of auditory experience. They found that the superior temporal cortex was recruited for the 2-back task but not the control task in only the DHH group, regardless of the linguistic nature of the stimuli. They hypothesized that the superior temporal cortex, which is an auditory processing area in typically hearing people, underwent a cross-modal reorganization to then subserve domain-general WM in DHH people, allowing for a reduced cortical recruitment of the parietal and frontal regions, which are typically recruited for WM in typically hearing people. These neural changes in the DHH group were accompanied by faster reaction times (but no change in accuracy) for the DHH group as compared to the hearing groups for only the 2-back task, but not for the control task (detecting whether any dots in the display were yellow). However, it is important to note that each item in the 2-back task (either a BSL sign or an object) was a dynamic point-light display, which elicits spatial motion processing. Accordingly, Cardin et al. (2018) interpreted the reaction time difference as more in line with previous studies showing a DHH advantage on tasks involving dynamic visuospatial information, rather than serial recall per se (Keehner & Atkinson, 2006).

### 1.3. Current Study

This study is the first to systematically examine the performance of DHH children on the *n*-back task. We examined the longitudinal changes in performance on visual 1-back and 2-back tasks among a sample of 103 DHH signing children aged 7–11 years across a period of two years. The use of an accelerated longitudinal design allowed us to cover an age range of 7 to 13 years. In this first longitudinal study of WM development in DHH children, we examined the developmental changes in their n-back WM performance and determined whether their performance on these tasks was predicted by their family background and language ability. We predicted that their task performance would improve across the age range tested, with larger age-related gains for the children from deaf families compared to those from hearing families (reflecting the positive effect of strong early language exposure). By including a standardized measure of sign language ability—the ASL Receptive Skills test—we further sought to determine whether any effect of the parental hearing status was mediated by language ability.

## 2. Materials and Methods

### 2.1. Participants

The participants (N = 103) were students who studied at one of five bilingual–bicultural schools for the deaf across the United States. All the schools employed American Sign Language (ASL) as the primary language of instruction and interaction, and English as the primary written language. The participants were recruited through the schools’ communications with parents, including social media posts, letters, emails, and at school events or after-school pickups. The study was approved by the Institutional Review Board of Rochester Institute of Technology and by the research review boards of all the participating schools.

The participants were between 7 and 11 years of age upon inclusion into the study. To be included, they had to have some degree of diagnosed hearing loss as well as their primary language of instruction being ASL. The participants were excluded if they had documented learning or intellectual disabilities or mobility issues that would preclude them from performing the tasks. A reported ADHD diagnosis was not grounds for exclusion; 10 participants had a reported ADHD diagnosis. Those 10 children did not differ from the rest of the sample in their demographics or task performance.

Being enrolled in bilingual schools for the deaf, the children included in this study shared a commonality of having sustained language input in at least one accessible language from an early age up until when the study was conducted. The children were assessed with the following language and academic measures (detailed descriptions under the *Measures* section): ASL comprehension (ASL-RST), a measure of spoken English ability (OWLS-LC), self-reported hearing level, and nonverbal IQ (KBIT-2). The demographic data for all the children are reported in Table 1; the language assessments, nonverbal IQ, and audiological data are presented in Table 2.

### 2.2. Measures

#### 2.2.1. N-Back Working Memory Task

**Figure 1:**
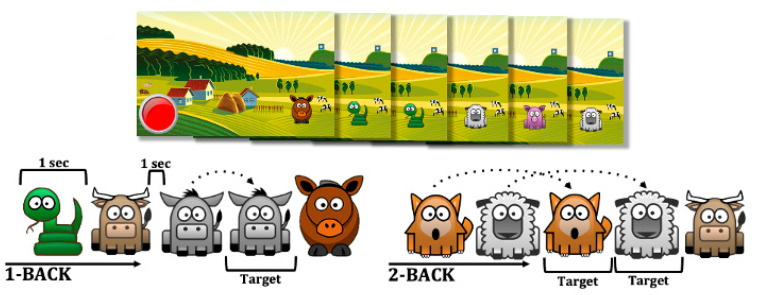
Visual representation of the n-back task employed in this study.

The *n*-back experiment used in this study was developed in-house with the Paradigm software program for building and running psychological experiments (Perception Research Systems, 2007). The experiment constituted two tasks that were wholly identical besides the *n*, which dictated which items were the targets: first the participants performed a 1-back task, then a 2-back task. In the 1-back task, an item was a target if it was identical to the item immediately prior; in the 2-back task, an item was a target if it was identical to the item appearing before the prior item. The items were cartoon images of “farm animals” (with 11 different types of animals), which appeared in one location on the screen, set against a cartoon “farm” background (see Figure 1).

A repeatable practice block preceded each experimental block, with the 2-back practice block occurring after the 1-back experimental block. The experimenter encouraged the child to repeat the practice block if they saw that the child had not yet understood the instructions; this was common for the 2-back task. Each experimental block contained a randomized order of items, which changed upon each iteration of the block. The blocks ended after 10 targets appeared (regardless of whether the child correctly responded to them or not) or after 50 total trials, whichever occurred first. Nine percent of the 1-back tasks and eight percent of the 2-back tasks reached the maximum of 50 total trials without 10 targets appearing. Each item appeared for 1000 ms, with inter-item intervals of 1000 ms.

#### 2.2.2. OWLS-II Listening Comprehension Subscale

Their spoken English receptive skills were assessed using the OWLS-II Listening Comprehension Subscale (Carrow-Woolfolk, 1995). For the OWLS-II LC, children listen to a spoken sentence and select the best-matching picture from four possible options. The items assess lexical/semantic, syntactic, pragmatic, and supralinguistic comprehension. Since the researcher was deaf, the items were presented as individual video files on a laptop, for which a hearing colleague spoke each item. The laptop was positioned in front of each child with a stereo speaker placed in front of the laptop; the response book was next to the laptop. Each child was prompted to watch each video without touching the table or speaker to feel the vibrations (a common request). The children wore their hearing aids during the test only if they reported regular hearing aids use. After completion of the test, the children were reassured that their performance on the test was not critical (to minimize the stereotype threat or associated negative feelings). The raw scores are reported in lieu of standardized scores because no standardized population norms exist for the test and the minimum standardized score of 40 obscures much of the variability in the raw scores.

#### 2.2.3. ASL Receptive Skills Test

ASL receptive skills were assessed using the ASL Receptive Skills Test (ASL-RST; Enns et al., 2013). The ASL-RST comprises 42 items, for each of which an ASL sentence is given and the child selects the best-matching illustration from four possible options. The ASL-RST was designed for use with children aged 3–12 years.

#### 2.2.4. Kaufman Brief Intelligence Test, Second Edition (Matrices)

Nonverbal intelligence (NVIQ) was assessed using the Matrices subtest of the Kaufman Brief Intelligence Test, Second Edition (KBIT-2), which is the only nonverbal subtest within the KBIT-2 (Kaufman & Kaufman, 2004). Each item contains a visual puzzle with an incomplete segment, and six possible answers. Only the correct answer will perfectly complete the pattern presented in the puzzles, and the items increase in difficulty, either by expanding the matrix and/or by presenting more complicated patterns. All the KBIT-2 scores were converted to standardized scores for the analyses in this study.

### 2.3. Procedure

All the testing was performed at the schools, either during or after the school day, as determined by the schools’ testing or administrative departments and/or parental preference. On rare occasions, testing was performed in the home upon parental request. The researcher visited each school a total of four times, with each visit lasting 2–4 weeks. They met with each child participating in the study for 1–2 testing sessions per visit (described below). The researcher who administered the tests was deaf and ASL-fluent. The testing took place while the researcher and child were alone in a distraction-free room. The instructions were administered in ASL.

Each child received a battery of attention, language, and cognition tests that comprised a larger project; the *n*-back task reported here was one of four cognitive tests given during each of the four visits. The tests were all given during a one-hour session upon every visit, for a maximum of four cognitive assessment sessions per child. Due to the logistical limitations of travel and testing, the four visits for data collection were not spaced out uniformly (time between visits 1–2: *M* = 248 days, σ = 14.8; visits 2–3: *M* = 172 days, σ = 7.33; visits 3–4: *M* = 236 days, σ = 40.2). Because of this, as well as the staggered ages of the participants included in the study, all the longitudinal models were calculated using the centered *age* as the time parameter rather than the number of the visit.

All the visits contained at least one session, during which cognitive tests were administered. For each child, a second session was administered during the visit that was the closest possible to the child’s 10th birthday. Language and NVIQ measures were obtained during this second session, which was administered at least 24 h after a cognitive assessment and no more than 2 weeks following a cognitive assessment. The order of the assessments was counterbalanced in all the sessions, except for the language measures. The ASL and spoken English assessments were always given at the end of the session to avoid a potential stereotype threat from administering tests of spoken and signed language to DHH children in a bilingual–bicultural environment.

### 2.4. Apparatus

The n-back task was administered using *Paradigm* experimental software, running on a Getac F110 tablet computer with an integrated 11.6″ HD touchscreen (resolution: 1366-by-768 pixels; brightness: 800 nits) and a Windows 7 operating system. The responses were recorded via touchscreen. The language tests were administered using a Dell Latitude E7470 14″ laptop; for the audio during the spoken language tests, the laptop was connected to a Bose SoundLink Mini II stereo speaker placed on the table in front of the participant.

### 2.5. Statistical Analyses

All the statistical analyses were conducted using R version 4.4.3 running within Posit R Studio version 2024.12.1+563. Linear mixed models were performed using the lme4 toolbox (version 1.1-35.5; Bates et al., 2015). The full analysis script is available on the Open Science Framework—see the Data Availability Statement.

## 3. Results

### 3.1. Model Building

We took a linear mixed model building approach following the recommendations of Meteyard and Davies (2020). The model specifications and measures of fit are provided in Table 3. For all the models, the dependent measure was the proportion of correct responses. All the models were based upon 708 observations made of 103 children.

### 3.2. Base Growth Model

We started with an intercept-only model in which the intercepts were free to vary for each child. This base model reflected an overall average performance accuracy of 54.5% across the testing sessions, which was significantly better than zero (t = 45.41; *p* < 0.001). We then added the age as a fixed within-subjects effect and allowed for both the intercepts and slopes to vary to create a linear growth model. However, the correlation between the random effects was −1, indicating overfitting and an inability to reliably estimate the random effects of both the intercept and slope in the same model. The random effect of the slope was therefore removed to yield a random intercepts-only linear growth model that significantly improved the fit of the model to the data ($\chi^{2}$ = 56.10; df = 1; *p* < 0.001). This model revealed that the performance accuracy improved as the children grew older, by 5.6% per year. Next, we added a quadratic age term to see whether modeling growth as a nonlinear predictor had a significant advantage over the linear growth model. The quadratic model did not provide a better fit than the linear model ($\chi^{2}$ = 0.60; df = 1; *p* > 0.05) and the age squared was not a significant predictor—the nonlinear growth model was therefore discarded. The base linear growth model with random intercepts is reported in Table 4.

### 3.3. Fixed Effects and Interaction Models

The base growth model served as the basis for the models that sought to determine the effects of task difficulty (1-back vs. 2-back) and family background (deaf family vs. hearing family) on the performance accuracy.

#### 3.3.1. Task Difficulty

We started by adding the task difficulty (1-back vs. 2-back) as a main effect, which resulted in a significantly improved model fit ($\chi^{2}$ = 521.73; df = 1; *p* < 0.001). Starting from an intercept of 73.4%, the accuracy improved by 6.4% per year (t = 10.12; *p* < 0.001), and moving from a 1-back to a 2-back task resulted in a drop in accuracy of 37.7% (t = −28.77; *p* < 0.001). Next, we added an interaction term to determine whether the significant effect of task difficulty interacted with the age. The significance of the main effects remained, but the interaction term was not statistically significant (t = −1.49; *p* = 0.136), and so the interaction model was discarded. The model for task difficulty is reported in Table 5.

#### 3.3.2. Effect of Family Background

**Figure 2:**
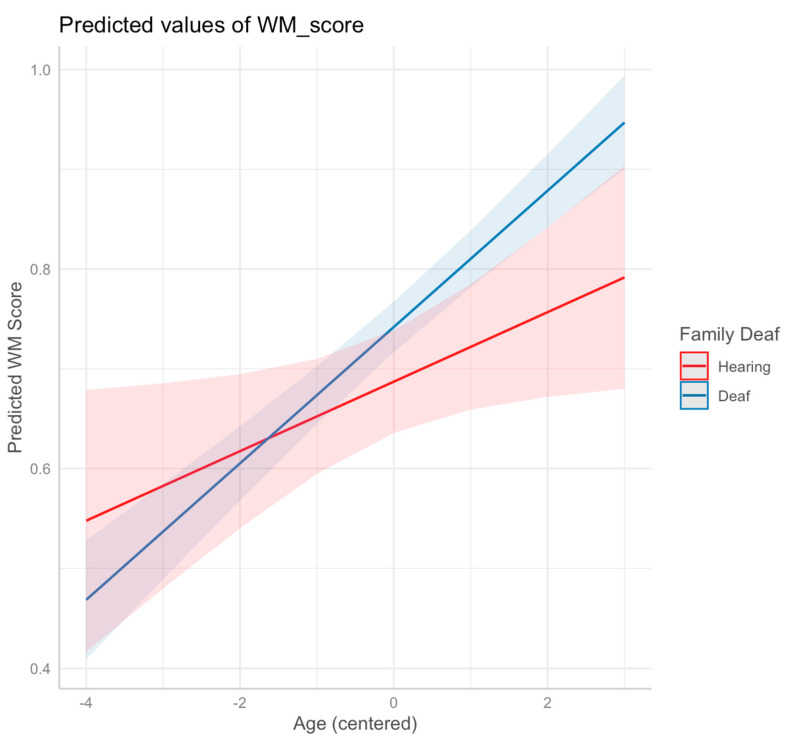
Predicted values of *n*-back working memory (WM) performance as a function of age, separated by family background (parental hearing status). The shaded areas represent the 95% confidence interval for the slope magnitude.

Our next step was to add the family background to the model as a fixed effect, to see whether the parental hearing status—a proxy for early access to natural language—affected the working memory performance. While adding the family background only marginally improved the model fit ($\chi^{2}$ = 3.19; df = 1; *p* = 0.074), it revealed a marginally significant effect of family background on task performance (t = 1.81; *p* = 0.073). We next added an interaction term to the model to explore how the family background interacted with growth over time; this was our final model and is reported in full in Table 6. The final model represented a marginally significant increase in the goodness of fit ($\chi^{2}$ = 3.70; df = 1; *p* = 0.054). The main effects of age (t = 2.18; *p* = 0.030) and task difficulty (t = −28.80; *p* < 0.001) remained statistically significant, with a marginally significant main effect of family background (t = 1.98; *p* = 0.051) and a marginally significant interaction between family background and age (t = 1.93; *p* = 0.055). The slopes for the two groups of children are shown in Figure 2, with the descriptive statistics reported in Table 7.

### 3.4. Mediating Effect of ASL

The DHH children from DHH families had significantly higher ASL Receptive Skills Test scores than did those from hearing families (see Table 2). We therefore fitted an ordinary least square fixed effects model to predict the 2-back working memory accuracy from the centered age, family background (deaf parents vs. hearing parents), and centered ASL-RST standard scores. This model revealed an intercept of 32.5%, with the significant main effects of age (t = 2.78; *p* = 0.006; estimate = 10.7%) and ASL-RST score (t = 2.23; *p* = 0.028; estimate = 1.0%). Of particular interest, the effect of family background (t = −0.21; *p* = 0.835; estimate = −1.0%) and its interaction with age (t = −1.64; *p* = 0.104; estimate = −7%) were no longer statistically significant. Therefore, after controlling for ASL receptive skills, the DHH children from DHH families no longer presented with steeper growth trajectories for growth in working memory performance. This suggests that the effect of family background was mediated by the performance on the ASL receptive skills measure.

## 4. Discussion

In this paper, we sought to assess the performance of DHH children on a task that measures working memory (WM) while minimizing the ability of the children to use heuristic shortcuts, which may lend an advantage to the children who use a spoken language. While such an advantage is most pertinent when comparing deaf versus hearing children, it may also confound the results within an entirely DHH group (such as in this study) if some individuals within this group have stronger spoken language skills than others. We chose the *n*-back task, which requires children to recall items presented prior to the current item. Since the children could not know which items were relevant (i.e., the first item of a matching pair) until after the appearance of the next or second-to-next paired item, we argue that this task limits the use of heuristic shortcuts to boost task performance. To assess the development of WM skills, we collected data from each child across multiple time points. We also examined the influence of early childhood language environment and skill by including the family background and the child’s performance on an ASL receptive skills assessment in our models.

We found that, as expected, the 2-back task was more challenging than the 1-back task, but that this was not influenced by age. The children in our sample individually demonstrated an improvement in performance across the ages of 7–13 years, indicating that our WM task captured the development in WM capacity for this age range. When assessing the influence of language on WM capacity, we found that the DHH children with DHH parents demonstrated accelerated improvement in WM capacity. We also found that this relationship was mediated by ASL skill.

We present two main findings in this paper. First, we recommend the *n*-back task as a possible alternative to tasks, such as serial span tasks, when assessing WM capacity in children who do not use a spoken language as their first language. Serial span tasks allow for articulatory-based heuristic strategies, which may bias such measures against signing children. This was suggested by Hall and Bavelier (2010), who proposed the *multiple coding hypothesis*: speakers depend on the articulatory loop much more than signers for encoding information into WM, whereas signers rely on more distributed coding across signed phonological, visuospatial, or episodic processes. They suggested that instead of truly reflecting serial WM capacities, serial span tasks reward a reliance on verbal chunking and the rote rehearsal of information, which are articulatory heuristic strategies that give hearing speakers an advantage over DHH signers on the ordered recall of fixed spans. However, as we did not explicitly compare the *n*-back task with serial span tasks, we recommend that future research compare the performance of DHH and hearing children on each type of task to determine whether disparities in serial span performance are replicated on *n*-back tasks.

Second, we suggest that our data support the importance of a strong foundation of accessible language instruction for the development of cognitive skills, such as working memory. Specifically, we emphasize that signed language instruction supports the development of cognitive skills often thought to be specifically scaffolded by *spoken* input, such as serial STM. One difficulty inherent in comparing DHH children with DHH parents to DHH children with typically hearing parents is that these two populations of children vary in multiple ways. A key difference is in the timing and quality of early accessible language input. Children with DHH signing parents are typically exposed to phonologically and syntactically rich ASL from birth, whereas hearing parents who choose to sign with their DHH infants will (i) initiate that language input after the detection of hearing loss and (ii) will usually be learning the language at the same time as they are using it to communicate with their deaf infant and have beginner-to-intermediate language learner ability (Pontecorvo et al., 2024). The significance of this latter difference has recently been called into question by research demonstrating similar rates of ASL vocabulary acquisition in DHH children regardless of the hearing status of their parents if exposure starts prior to 6 months of age (Caselli et al., 2021). However, for some DHH children with typically hearing parents, early communicative interactions may be disrupted, resulting in executive function deficits (Morgan & Dye, 2020), increased parenting stress (Quittner et al., 2010), and differences in parent–child interactions (see Curtin et al., 2021 for a review). In the current study, controlling for ASL receptive skills removed the DoD versus DoH parent group differences in the growth of working memory performance. However, assuming that ASL receptive skills are correlated with these factors due to the influence of early communicative interactions, we should be cautious in focusing any interventions solely on language instruction. The timing and quality of language input, as well as the resulting parent–child interactions driven by that input, are likely to play crucial roles in scaffolding cognitive development in DHH infants and young children.

## Figures and Tables

**Table 1:** Demographic characteristics of DHH sample.

	DHH with Hearing Parents	DHH with DHH Parents
Sample size (n)	18	85
Age (years), M (SD)	9.73 (1.48)	9.73 (1.35)
Parental education ^1^, M (SD)	3.12 (1.31)	3.94 (1.12) *
Racial distribution ^2^	7/10/1	58/26/1
Gender distribution ^3^	6/12/0	43/41/1

^1^ 1 = did not graduate high school; 2 = high school graduate; 3 = some college; 4 = 4-year college graduate; 5 = graduate degree. ^2^ White/Black, Indigenous and people of color/did not respond. ^3^ Male/female/transgender or non-binary. * *p* < 0.05.

**Table 2:** Linguistic, NVIQ, and audiological characteristics of DHH sample.

	DHH with Hearing Parents	DHH with DHH Parents
Sample size (n)	18	85
OWLS II Listening Comprehension (raw), M (SD)	34.0 (33.5)	19.3 (25.9)
ASL Receptive Skills Test (standard), M (SD)	101.5 (4.41)	105.7 (3.39) *
KBIT-2 Matrices (standard), M (SD)	106.0 (8.76)	102.8 (15.9)
Self-reported hearing level distribution ^1^	1/4/4/8/1	4/19/15/44/3

^1^ 1 = mild; 2 = moderate; 3 = severe; 4 = profound; 5 = no response. * *p* < 0.05.

**Table 3:** Model specifications and measures of fit for linear mixed models.

Model Name	Nested Model	Fixed Effects	Random Effects		Model Fit				LRT Test Against Nested Model	
			Child		AIC	BIC	LL	df	df	$\chi^{2}$
Intercept only		-	Intercept	-	214.2	227.9	−104.1	705		
Linear growth	Intercept only	Age	Intercept	Slope	Cannot reliably estimate both random effects					
Linear growth, no random slope	Intercept only	Age	Intercept	-	160.1	178.4	−76.1	704	1	56.10 ***
Quadratic growth, no random slope	Linear growth, no random slope	Age + (Age *Age)	Intercept	-	161.5	184.4	−75.8	703	1	0.60
Linear growth with task difficulty	Linear growth, no random slope	Age + Task Difficulty	Intercept	-	−359.6	−336.8	184.8	703	1	521.73 ***
Linear growth with task difficulty	Linear growth with task difficulty	Age + Task Difficulty + Age * Task Difficulty	Intercept	-	−359.8	−332.4	185.9	702	1	2.25
Linear growth with task difficulty with deaf family	Linear growth with task difficulty	Age + Task Difficulty + Deaf Family	Intercept	-	−360.8	−333.4	186.4	702	1	3.19 ^+^
Linear growth with deaf family with task difficulty	Linear growth with task difficulty with deaf family	Age + Task Difficulty + Deaf Family + Age * Deaf Family	Intercept	-	−362.5	−330.5	188.2	701	1	3.70 ^+^

*** *p* < 0.001; ^+^ *p* < 0.10.

**Table 4:** Base growth model.

Fixed Effects					
	β estimate	SE	95% CI	t	*p*
Intercept	0.546	0.010	0.525–0.566	52.04	<0.0001
Age	0.056	0.007	0.042–0.070	7.81	<0.0001
Random Effects					
	Variance		SD	Correlation	
Child (intercept)	0.0008		0.0288	–	
Model Fit					
R^2^	Marginal			Conditional	
	0.083			0.094	
Key: *p*-values for fixed effects calculated using Satterthwaite’s approximations. Confidence intervals calculated using Wald method.					
Model equation:		$y_{ij}=\beta_{0}+\beta_{1}x_{ij}+b_{0j}+\varepsilon_{ij}\;$			

**Table 5:** Base growth model with fixed main effect of task difficulty.

Fixed Effects					
	β estimate	SE	95% CI	t	*p*
Intercept	0.734	0.012	0.710–0.758	59.23	<0.0001
Age	0.064	0.006	0.052–0.077	10.12	<0.0001
Task Difficulty	−0.378	0.013	−0.403–−0.351	−28.77	<0.0001
Random Effects					
	Variance		SD	Correlation	
Child (intercept)	0.0067		0.0822	–	
Model Fit					
R^2^	Marginal			Conditional	
	0.545			0.627	
Key: *p*-values for fixed effects calculated using Satterthwaite’s approximations. Confidence intervals calculated using Wald method.					
Model equation:		$y_{ij}=\beta_{0}+\beta_{1}x_{ij}+{\beta 2}^{Τ}d_{ij}+b_{0j}+\varepsilon_{ij}\;$			

**Table 6:** Base growth model with fixed main effects of task difficulty and family background, with interaction term between age and family background.

Fixed Effects					
	β estimate	SE	95% CI	t	*p*
Intercept	0.687	0.026	0.636–0.738	26.22	<0.0001
Age	0.035	0.016	0.003–0.066	2.18	0.030
Task Difficulty	−0.377	0.013	−0.403–−0.351	−28.80	<0.0001
Family Background	0.055	0.028	0.000–0.109	1.98	0.051
Interaction: Family Background with Age	0.036	0.017	−0.001–0.068	1.93	0.055
Random Effects					
	Variance		SD	Correlation	
Child (intercept)	0.0061		0.0784	–	
Model Fit					
R^2^	Marginal			Conditional	
	0.552			0.628	
Key: *p*-values for fixed effects calculated using Satterthwaite’s approximations. Confidence intervals calculated using Wald method.					
Model equation:	$y_{ij}=\beta_{0}+\beta_{1}x_{ij}+\beta_{2}f_{j}+\beta_{3}(x_{ij}\;\cdot \;f_{j})\;{+\beta 4}^{Τ}d_{ij}\;{+b}_{0j}+\varepsilon_{ij}\;$				

**Table 7:** Descriptive statistics for working memory performance (accuracy as percent correct) as function of task difficulty and family background. Centered age effects are shown by separating data into quartiles.

Task Difficulty	Family Background	Age Quartile	Mean Centered Age	WM Accuracy (M)	WM Accuracy (SD)	N
1-back	Hearing parents	1	–1.89	63.1	24.1	16
		2	–0.44	65.0	23.5	17
		3	0.60	70.0	24.5	12
		4	1.86	72.9	18.8	10
	Deaf parents	1	–1.98	59.5	21.8	73
		2	–0.50	74.7	17.1	71
		3	0.61	78.2	16.1	77
		4	1.80	84.9	15.5	78
2-back	Hearing parents	1	–1.89	23.1	17.0	16
		2	–0.44	26.5	19.0	17
		3	0.60	34.4	22.1	12
		4	1.86	43.2	19.8	10
	Deaf parents	1	–1.99	27.8	19.7	72
		2	–0.51	34.2	20.7	72
		3	0.60	36.2	18.0	76
		4	1.79	47.5	21.8	79

Note. Age quartiles are based on the distribution of the centered age across the full sample. Negative values reflect participants below the sample mean age, and positive values reflect those above.

## Data Availability

A cleaned and tidied dataset and the R code required to replicate and extend the reported analyses are available at https://osf.io/kpvuz/ (accessed on 1 July 2025).

## Abbreviations

The following abbreviations are used in this manuscript:

DHH	Deaf or hard of hearing
ASL	American Sign Language
BSL	British Sign Language
DoD	Deaf child(ren) with at least one deaf parent(s)
DoH	Deaf child(ren) with hearing parents
WM	Working memory
STM	Short-term memory
ADHD	Attention-deficit/hyperactivity disorder
CI	Cochlear implant
NVIQ	Nonverbal intelligence
ASL-RST	American Sign Language Receptive Skills Test
OWLS-LC	Oral and Written Language Scales II, Listening Comprehension Subtest
KBIT-2	Kaufman Brief Intelligence Test, Second Edition, Matrices Subtest
M (SD)	Mean (standard deviation)

## References

[B1-behavsci-15-00919] Baddeley A. D., Hitch G., Bower G. H. (1974). Working memory. Psychology of learning and motivation.

[B2-behavsci-15-00919] Bates U., Maechler M., Bolker B., Walker S. (2015). Fitting linear mixed-effects models using lme4. Journal of Statistical Software.

[B3-behavsci-15-00919] Bavelier D., Newport E. L., Hall M., Supalla T., Boutla M. (2008). Ordered short-term memory differs in signers and speakers: Implications for models of short-term memory. Cognition.

[B4-behavsci-15-00919] Bellugi U., Klima E. S., Siple P. (1974). Remembering in signs. Cognition.

[B5-behavsci-15-00919] Cardin V., Rudner M., De Oliveira R. F., Andin J., Su M. T., Beese L., Woll B., Rönnberg J. (2018). The organization of working memory networks is shaped by early sensory experience. Cerebral Cortex.

[B6-behavsci-15-00919] Carrow-Woolfolk E. (1995). Oral and written language scales: Listening comprehension and oral expression (OWLS).

[B7-behavsci-15-00919] Caselli N., Pyers J., Lieberman A. M. (2021). Deaf children of hearing parents have age-level vocabulary growth when exposed to American sign language by 6 months of age. The Journal of Pediatrics.

[B8-behavsci-15-00919] Cleary M., Pisoni D. B., Geers A. E. (2001). Some measures of verbal and spatial working memory in eight- and nine-year-old hearing-impaired children with cochlear implants. Ear and Hearing.

[B9-behavsci-15-00919] Cowan N. (2017). The many faces of working memory and short-term storage. Psychonomic Bulletin & Review.

[B10-behavsci-15-00919] Curtin M., Dirks E., Cruice M., Herman R., Newman L., Rodgers L., Morgan G. (2021). Assessing parent behaviours in parent–child interactions with deaf and hard of hearing infants aged 0–3 years: A systematic review. Journal of Clinical Medicine.

[B11-behavsci-15-00919] Delcenserie A., Genesee F., Champoux F. (2024). Exposure to sign language prior and after cochlear implantation increases language and cognitive skills in deaf children. Developmental Science.

[B12-behavsci-15-00919] Engle R. W., Tuholski S. W., Laughlin J. E., Conway A. R. A. (1999). Working memory, short-term memory, and general fluid intelligence: A latent-variable approach. Journal of Experimental Psychology. General.

[B13-behavsci-15-00919] Enns C. E., Zimmer K., Boudreault P., Rabu S., Broszeit C. (2013). American sign language receptive skills test.

[B14-behavsci-15-00919] Goodwin C., Carrigan E., Walker K., Coppola M. (2022). Language not auditory experience is related to parent-reported executive functioning in preschool-aged deaf and hard-of-hearing children. Child Development.

[B15-behavsci-15-00919] Hall M. L., Bavelier D. (2010). Working memory, deafness, and sign language. The Oxford handbook of deaf studies, language, and education.

[B16-behavsci-15-00919] Hall M. L., Eigsti I.-M., Bortfeld H., Lillo-Martin D. (2018). Executive function in deaf children: Auditory access and language access. Journal of Speech, Language, and Hearing Research.

[B17-behavsci-15-00919] Jamsek I. A., Kronenberger W. G., Pisoni D. B., Holt R. F. (2022). Executive functioning and spoken language skills in young children with hearing aids and cochlear implants: Longitudinal findings. Frontiers in Psychology.

[B18-behavsci-15-00919] Kaufman A. S., Kaufman N. L. (2004). Kaufman brief intelligence test.

[B19-behavsci-15-00919] Keehner M., Atkinson J., Pickering  S. J. (2006). Working memory and deafness: Implications for cognitive development and functioning. Working memory and education.

[B20-behavsci-15-00919] Kronenberger W. G., Beer J., Castellanos I., Pisoni D. B., Miyamoto R. T. (2014). Neurocognitive risk in children with cochlear implants. JAMA Otolaryngology–Head & Neck Surgery.

[B21-behavsci-15-00919] Marschark M., Kronenberger W. G., Rosica M., Borgna G., Convertino C., Durkin A., Machmer E., Schmitz K. L. (2016). Social maturity and executive function among deaf learners. The Journal of Deaf Studies and Deaf Education.

[B22-behavsci-15-00919] Marshall C., Jones A., Denmark T., Mason K., Atkinson J., Botting N., Morgan G. (2015). Deaf children’s non-verbal working memory is impacted by their language experience. Frontiers in Psychology.

[B23-behavsci-15-00919] McCreery R. W., Walker E. A. (2022). Variation in Auditory experience affects language and executive function skills in children who are hard of hearing. Ear and Hearing.

[B24-behavsci-15-00919] Meteyard L., Davies R. A. I. (2020). Best practice guidance for linear mixed-effects models in psychological science. Journal of Memory and Language.

[B25-behavsci-15-00919] Morgan G., Dye M. W. G., Marschark M., Knoors H. (2020). Executive functions and access to language: The importance of intersubjectivity. The Oxford handbook of deaf studies in learning and cognition.

[B26-behavsci-15-00919] Pelegrina S., Lechuga M. T., García-Madruga J. A., Elosúa M. R., Macizo P., Carreiras M., Fuentes L. J., Bajo M. T. (2015). Normative data on the n-back task for children and young adolescents. Frontiers in Psychology.

[B27-behavsci-15-00919] Perception Research Systems (2007). Paradigm stimulus presentation.

[B28-behavsci-15-00919] Pisoni D. B., Cleary M. (2003). Measures of working memory span and verbal rehearsal speed in deaf children after cochlear implantation. Ear and Hearing.

[B29-behavsci-15-00919] Pisoni D. B., Geers A. E. (2000). Working memory in deaf children with cochlear implants: Correlations between digit span and measures of spoken language processing. Annals of Otology, Rhinology & Laryngology.

[B30-behavsci-15-00919] Pontecorvo E., Mitchiner J., Lieberman A. M. (2024). Hearing parents as sign language learners: Describing and evaluating the ASL skills of parents learning ASL with their deaf children. Journal of Multilingual and Multicultural Development.

[B31-behavsci-15-00919] Quittner A. L., Barker D. H., Cruz I., Snell C., Grimley M. E., Botteri M., Eisenberg L., Luxford W., Johnson K., Martinez A., DesJardin J., Visser-Dumont L., Ambrose S., Stika C., Gillinger M., Niparko J., Chinnici J., Francis H., Bowditch S., Bayton P. (2010). Parenting stress among parents of deaf and hearing children: Associations with language delays and behavior problems. Parenting.

